# *Bacillus cereus* strain L4J2, isolated from Mexican soil, carries multiple genes for biotechnological application

**DOI:** 10.1128/mra.01180-24

**Published:** 2025-04-01

**Authors:** Jemima Rangel-Heredia, Cristian A. Flores-Cruz, César I. Hernández-Vásquez, Saúl A. Martínez-Morales, Luis J. Galán-Wong, Benito Pereyra-Alférez

**Affiliations:** 1 Facultad de Ciencias Biológicas, Instituto de Biotecnología, Universidad Autónoma de Nuevo León 250520, San Nicolás de los Garza, Nuevo Leon, Mexico; Rochester Institute of Technology, Rochester, New York, USA

**Keywords:** bacterial genome, computational biology

## Abstract

*Bacillus cereus* L4J2, isolated from Mexican soil and initially identified as *Bacillus thuringiensis* due to its crystalline inclusions with mosquito activity, belongs to the *Bacillus cereus* species group. Its genome is larger than other *Bacillus cereus* strains and contains genes with significant biotechnological applications.

## ANNOUNCEMENT

The *Bacillus cereus* strain L4J2 was isolated from agricultural soil collected in the north of Mexico. The isolation was performed using the standard methodology described by Travers et al. (1), which involves heat treatment and selective media to isolate spore-forming *Bacillus* species. This strain is a Grampositive and sporulated bacterium. Preliminary bioassay results revealed that crystal inclusion proteins from the *B. cereus* strain L4J2 showed lethal activity against larvae of *Aedes aegypti*.

For the whole-genome sequencing of *Bacillus cereus* L4J2, cells were obtained from our laboratory cryostock and grown in 50 mL of nutrient broth for 8 h at 35°C under constant agitation at 150 rpm. Culture was centrifuged, and the pellet was washed twice with sterile water. After that, the cells were shipped chilled to GeneWiz for total DNA extraction and library preparation. The whole genome was sequenced using Illumina MiSeq, which resulted in 43,414,186 paired-end reads of 300 bp with a coverage of 250.0×. For genome assembly and annotation, in brief, the sequence quality was determined with FastQC 0.12.1 (2). Then, low-quality sequences and adapters were removed with Trimmgalore 0.6.10 (3). Processed sequence reads were assembled with SPAdes 3.15.4 using k-mers of 21,35,55,127 with a phred-offset of 33 (4); the resulting contigs were annotated with BAKTA 1.9.1 (5) and with the NCBI Prokaryotic Genome Annotation Pipeline (PGAP version 6.8) using the best-placed reference protein set method (GeneMarkS-2+) (6, 7).

We identified a 7,649,903 bp genome with an N50 value of 318,863 bp, an L50 of 7, and an L90 of 24, with a total of 370 contigs, 6,165 open reading frames (ORF), 35.23% GC content, 125 tRNA operons, 23 rRNAs, and 31 noncoding RNAs (ncRNAs).(Table 1) An average nucleotide identity (ANI) (8) analysis was performed during the GenBank submission process to determine identity with the type of genome of *Bacillus cereus*. Presenting a 95.83% genome identity with *Bacillus cereus* FORC_047 and 95.53% with *Bacillus cereus* ATCC 14579, JSpeciesWS 4.2.1 (9) which uses blast +2.11.0 (10) the parameter for this analysis was comparing the genome with both *Bacillus cereus* and *Bacillus thuringiensis* reference genomes. The genome sequence was analyzed to identify genes of biotechnological interest (11–14). In this sense, the BAKTA tool revealed that *B. cereus* L4J2 carries genes encoding Cry 11Aa, Cry 60 Aa Cry 10 Aa, Cyt 1Aa, Cyt 2Ba, Cyt K2, Nhe subunit A, B, and C, Hbl components L1, L2, and HBL A and B.

**TABLE 1:** Summary of the genomic characteristics of *Bacillus cereus* L4J2 strain

	Genome size (bp)	GC%	N50 (bp)	Contigs
*B. cereus* L4J2	7,649,903	35.23	318,863	370

## Data Availability

The *Bacillus cereus* strain L4J2 whole-genome shotgun sequence will be deposited in GenBank under the accession number JBFBPH000000000 and BioProject number PRJNA1130484. The raw sequence files will be available under SRA number SRR29744432.
